# Neuroimaging Endpoints in Amyotrophic Lateral Sclerosis

**DOI:** 10.1007/s13311-016-0484-9

**Published:** 2016-10-17

**Authors:** Ricarda A. L. Menke, Federica Agosta, Julian Grosskreutz, Massimo Filippi, Martin R. Turner

**Affiliations:** 1 Nuffield Department of Clinical Neurosciences, University of Oxford, Oxford, UK; 2 Neuroimaging Research Unit, Institute of Experimental Neurology, Division of Neuroscience, San Raffaele Scientific Institute, Vita-Salute San Raffaele University, Milan, Italy; 3 Hans-Berger Department of Neurology, Jena University Hospital, Jena, Germany; 4 Department of Neurology, San Raffaele Scientific Institute, Vita-Salute San Raffaele University, Milan, Italy

**Keywords:** Amyotrophic lateral sclerosis, motor neuron disease, magnetic resonance imaging, trial, biomarker.

## Abstract

Amyotrophic lateral sclerosis (ALS) is a progressive neurodegenerative, clinically heterogeneous syndrome pathologically overlapping with frontotemporal dementia. To date, therapeutic trials in animal models have not been able to predict treatment response in humans, and the revised ALS Functional Rating Scale, which is based on coarse disability measures, remains the gold-standard measure of disease progression. Advances in neuroimaging have enabled mapping of functional, structural, and molecular aspects of ALS pathology, and these objective measures may be uniquely sensitive to the detection of propagation of pathology *in vivo*. Abnormalities are detectable before clinical symptoms develop, offering the potential for neuroprotective intervention in familial cases. Although promising neuroimaging biomarker candidates for diagnosis, prognosis, and disease progression have emerged, these have been from the study of necessarily select patient cohorts identified in specialized referral centers. Further multicenter research is now needed to establish their validity as therapeutic outcome measures.

**Electronic supplementary material:**

The online version of this article (doi:10.1007/s13311-016-0484-9) contains supplementary material, which is available to authorized users.

## Introduction

Amyotrophic lateral sclerosis (ALS) is a neurodegenerative disease of the motor system and its associated neuronal networks. Pathologically it is characterized by cytoplasmic inclusions of ubiquitinated TAR DNA-binding protein 43 in degenerating upper motor neurons (UMNs) of the primary motor and frontotemporal cortices, and lower motor neurons (LMNs) of the brainstem nuclei and spinal cord anterior horns. The syndrome is heterogeneous and overlaps clinically, pathologically, and genetically with frontotemporal dementia (FTD). Progressive muscle weakness leads eventually to death, typically caused by respiratory insufficiency, with a median survival from symptom onset of only 2 to 3 years [1].

ALS is emerging as a final common pathway from multiple upstream pathological mechanisms [2]. Approximately 10% of all cases of ALS are associated with mutations in a single gene (*C9orf72*, *SOD1*, *TARDBP*, *FUS*), and asymptomatic carriers of such mutations offer a window into the earliest pathological changes [3]. To date, animal models have not been able to predict treatment response in humans, and there are no validated biomarkers for human ALS beyond the clinically supported diagnostic application of electromyography, which is only 60% sensitive. Riluzole, thought to work by suppressing glutamatergic activity, is the only disease-modifying treatment for ALS, despite decades of drug trials.

ALS symptoms typically begin in the distal limb or bulbar musculature, and typically spread to contiguous body regions clinically [4], outwards from an apparent focus of pathology in postmortem studies [5]. The diagnosis remains clinical, and based upon the coincidence of UMN and LMN signs in the same body regions [6]. The dominance of UMN *versus* LMN signs is variable, with extremes of UMN involvement termed primary lateral sclerosis and those of LMN involvement, termed progressive muscular atrophy. These extremes are both associated with slower rates of progression [7–9]. The clinical, pathological, and genetic overlap of ALS with FTD is an adverse prognostic factor [10].

The revised ALS Functional Rating Scale (ALSFRS-R), which is based on coarse disability measures driven by LMN dysfunction and remote from histopathological changes, remains the gold-standard measure of disease progression [11, 12]. The incorporation of objective UMN biomarkers into drug trials in ALS, such as transcranial magnetic stimulation [13] or cerebrospinal fluid (CSF) neurofilaments [14], and LMN electrophysiological measures, such as motor unit number estimation [15] and electrical impedance myography [16], have gained increased attention. As well as improved participant stratification, they may help to reduce trial length and costs by providing more objective and sensitive surrogate markers of slowed disease progression or proof of target engagement.

Histopathological stages of TAR DNA-binding protein 43-positive pathology based on postmortem ALS brains support concepts of prion-like connectomic spread of pathology in ALS [17–19]. Advanced brain imaging techniques such as magnetic resonance imaging (MRI) and positron emission tomography (PET) over the last 20 years have bridged the gap between basic histopathological and molecular science and *in vivo* structural and functional abnormalities observed in the brain and spinal cord [20]. This review will focus on their potential as surrogate markers for diagnosis, stratification and monitoring disease progression of ALS in the context of therapeutic trials.

## Overview: Neuroimaging Techniques

### MRI: The Basics

#### Structural MRI

T1-weighted structural MRI results in images with good tissue contrast (gray matter, white matter, CSF), and is the method of choice for the investigation of gray matter, with the added advantage that the respective sequences are readily available on clinical MRI scanners. The most basic analysis approach is to utilize the acquired images in order to outline a region-of-interest (ROI) known to be affected in a disease process and to determine the *volume* of this structure. Conveniently, a number of currently available analysis tools now allow automated segmentation of various cortical and subcortical brain structures [21]. These techniques result not only in quantitative volumetric measures, but can also reveal local differences in thickness and surface shapes of structures and provide *cortical thickness* and surface area measures [22]. Automated segmentation tools help to avoid labor-intensive manual delineation, reduce inter-rater variability, and usually delineate structures with good accuracy, although problems can arise in morphometrically highly unusual brains. In addition to ROI approaches, other automated postprocessing pipelines such as *voxel-based morphometry* (VBM) enable statistics on *gray matter density* maps on a whole-brain, voxel-by-voxel basis, and can provide information on regional atrophy patterns on a group level without the need for *a priori* assumptions [23, 24].

Derivatives of VBM, such as voxel-based intensitometry can quantify ALS *white matter* pathology [25], but the most commonly applied technique is *diffusion tensor imaging* (DTI), which utilizes acquisition sequences sensitive to Brownian motion of water in fiber bundles [26]. DTI analysis is based on the observation that the displacement of water molecules within white matter is approximately elliptical, with greatest movement along axons, owing to the restrictions in perpendicular movement imposed by membranes. This behavior can be described with a diffusion tensor model, the mathematical formalism describing the elliptical displacement profile. The diffusion tensor is typically summarized by measures such as fractional anisotropy (FA), which describes how strongly directional the water displacement is within tissue, and mean diffusivity (MD), which reflects the average displacement distance independent of orientation. Intact white matter will restrict diffusion parallel to the main fiber direction (leading to higher FA and lower MD), whereas damage to white matter will cause diffusivity to be less restricted (i.e., lower FA and higher MD) [27]. Furthermore, analysis of directional diffusivities—parallel axial diffusivity (AD) and transverse or radial diffusivity (RD)—may provide additional information on the underlying mechanisms of white matter integrity loss. However, this simplistic interpretation of diffusion tensor metrics as measures of white matter integrity neglects the fact that diffusion tensor metrics are influenced by a variety of underlying microstructural causes [28], including axonal loss, demyelination, and swelling, as well as less orderly packing or the presence of multiple fiber populations (i.e., crossing [29] or “kissing” fibers) within a single MRI voxel. The insufficiency of the diffusion tensor model to describe fully multiple intravoxel fiber bundles has led to the development of novel data-acquisition approaches, such as high angular resolution diffusion imaging and diffusion spectrum imaging. Analysis of DTI metrics can be performed using ROI approaches, or whole-brain voxel-wise methods such as VBM-style analysis or tract-based spatial statistics (TBSS) [30]. A further development has been to apply graph theory to diffusion-weighted MRI data in order to build models of structural connectivity in brain disorders based on nodes and edges [31, 32].

#### Functional MRI

The most common functional MRI (fMRI) technique is based on the blood oxygen level-dependent (BOLD) response and the assumption that firing neurons cause locally increased energy demands, which result in a local increase of cerebral blood flow (CBF) and, to a lesser extent, local increases in cerebral oxygen metabolism and cerebral blood volume, in a way that creates a relative abundance of oxygenated hemoglobin in that area. Oxygenated and deoxygenated hemoglobin have different magnetic properties, which makes a change in their relative quantities detectable by MRI. Given enough repetitions of a task (e.g., an action or thought) performed in the scanner contrasted with rest periods, statistical methods can be used to determine the areas in the brain in which MR signal variations fluctuate in accordance with the task (*task-based fMRI*). A more recent development called *resting-state fMRI* enables the investigation of the coherence of regional MRI signals in the brain at rest (i.e., when a patient is not performing an explicit task). Even in the absence of external stimuli any brain region will have spontaneous fluctuations in BOLD signal, allowing the exploration of the brain’s functional organization and to investigate deviations from a healthy pattern in neurological disorders. *Resting-state functional connectivity* research has revealed a number of networks (representing networks of brain regions which show synchronous or *coherent* activity) that are consistently found in healthy subjects [33], and bear resemblance to networks shown to be involved in certain categories of tasks as revealed by task-based fMRI [34]. The 2 most popular methods for the analysis of resting state fMRI data are seed-based and independent component analysis (ICA). During seed-based analysis, data from *a priori*-defined voxels or ROI are used to calculate signal correlations with other voxels in the brain. This hypothesis-driven approach depends on a consistent way to define the seed within and between patients [35]. ICA, however, is a data-driven approach, which separates signal into nonoverlapping spatial components (i.e., networks of brain regions) according to their time courses. ICA-based approaches have the advantage of being fully automated and mostly observer-independent [36, 37]. However, data decomposition can slightly vary between runs and resulting components can be difficult to interpret and may not always clearly relate to brain structures of interest in a particular research context.

### Magnetic Resonance Spectroscopy

Magnetic resonance spectroscopy (MRS) is a means to explore the metabolite content of brain tissue *in vivo*. The proton is the nucleus with the highest nuclear magnetic resonance sensitivity and natural abundance in living tissue (>99.9 %). Proton MRS can robustly distinguish *N*-acetyl aspartate (NAA; a marker of neuronal integrity), choline (Cho; a marker of membrane integrity), and creatine (Cr; a chemical involved in energy metabolism). Furthermore, glutamate-related metabolites (glutamate and glutamine), as well as γ-amino butyric acid (GABA) can, depending on variables such as magnetic field strength, field homogeneity, and signal-to-noise ratio, be quantified separately or as a composite of glutamate, glutamine, GABA, and other metabolites in the brain.

### PET

PET is used to observe molecular metabolic processes in the brain using positron emitting radioisotopes (tracers) produced by a cyclotron. The tracer, injected peripherally, reaches the brain via a peripheral circulation injection. Subsequently, detectors in the PET scanner record simultaneous 180-degree pairs of gamma rays emitted as a consequence of positron-electron-annihilation, allowing 3-dimensional images that indicate the original radiotracer distribution in the brain to be reconstructed by computer analysis.

#### Activation PET

The most commonly used metabolic PET tracer for activation studies is ^18^F-fluorodeoxyglucose. Similar to fMRI studies, PET brain activation studies are based on the assumption that areas of high radioactivity are related to increased blood flow or, in this case glucose metabolism, and therefore surrogates for altered regional brain activity.

#### Ligand PET

Ligand PET allows visualization of neuroreceptor pools in the brain. For this purpose, tracers have been developed that are ligands for specific neuroreceptors. These may be expressed on neurons and so act as surrogates for neuronal loss, or reflect changes in neurotransmitter levels [38]. Another target has been benzodiazepine receptors expressed by microglia undergoing change from a resting phenotype to an activated phenotype in response to a wide variety of central nervous system insults, including ALS [39].

## Emerging Imaging Biomarkers in ALS

### Imaging for Diagnosis and Identification of Drug Targets

#### White Matter

The diagnostic delay in ALS (on average 1 year from symptom onset) is multifactorial and would not be completely addressed by the development of a diagnostic biomarker [40]. The detection of occult UMN involvement in clinically LMN-only cases might, however, increase trial recruitment [41]. DTI metrics have consistently demonstrated significant white matter alterations in the corticospinal tracts (CSTs) in ALS compared with healthy controls, reporting reduced FA [42–50] and increased MD and RD [51, 52]. DTI studies have specifically noted altered indices in the corpus callosum in ALS [53–55], as well extensive extramotor white matter involvement [56–58]. Asymptomatic carriers of pathogenic mutations in *SOD1* were also reported to have lower FA in the CSTs [59], though a result not replicated in a subsequent study [60]. Despite a consistently strong ALS DTI signature on the group level, a meta-analysis of CST FA measures revealed its diagnostic power to be modest even for the differentiation from healthy individuals, with a pooled sensitivity of 0.68 and a pooled specificity of 0.73 [61]. However, the heterogeneity of both the methodology and clinical phenotype of participants (especially cognitive impairment) may have been important factors.

#### Gray Matter

Compared with imaging measures reflecting white matter structural integrity, results of cross-sectional VBM studies in ALS have been somewhat inconclusive. Some studies have reported gray matter differences between patients and healthy controls (see, e.g., [50, 62, 63]), while others failed to demonstrate gray matter abnormalities (see, e.g., [64, 65]). However, a recent voxel-wise meta-analysis integrated results of 29 VBM studies comprising 638 patients with ALS and 622 healthy controls, to determine consistent gray matter abnormalities in ALS [66], and revealed disease-related atrophy mainly in the right precentral gyrus, the left Rolandic operculum, the left lenticular nucleus, and the right anterior cingulate and paracingulate gyri, in keeping with ALS as a pathological process extending beyond the motor system, even in the absence of overt cognitive deficits. Similarly, cortical thickness analyses have consistently revealed thinning of the precentral gyrus [67–69]. An initial study of the diagnostic performance of cortical thickness measurement alone against LMN disease mimics was, however, disappointing [70], but the combination of measures, either multimodal MRI [61, 71], or MRI and CSF measures [72], may be a strategy to improve accuracy.

Significant presymptomatic structural changes were also noted in carriers of C9orf72 hexanucleotide expansions, linked to the development of ALS and FTD [73, 74].

#### fMRI

In addition to structural abnormalities, fMRI studies have demonstrated increased cortical activity in patients with ALS performing motor tasks [75, 76], as well as aberrations in the BOLD response to cognitive tasks, such as antisaccade [77] or phonemic fluency tasks [78]. A number of fMRI studies investigating differences between patients with ALS and controls at rest have furthermore revealed functional connectivity abnormalities within the sensorimotor network, although some studies reported reduced functional connectivity in ALS [79–81], others increased motor network coherence [71, 82], and others a mixed picture [83, 84]. These inconsistencies are most likely driven by methodological differences and/or clinical and cognitive heterogeneity of patient samples. A graph theory, network approach to combined structural and function MRI datasets suggests that the 2 processes are coupled in ALS [85].

A shared signature of resting state functional connectivity changes involving the cerebellum was noted in both symptomatic sporadic patients and asymptomatic carriers of ALS-causing genetic mutations [86]. Replication of these findings in larger cohorts may reveal if functional connectivity changes are among the earliest detectable brain abnormalities in ALS.

#### PET

Preceding the wide availability of fMRI, the earliest activation PET studies also demonstrated widespread abnormalities of cerebral glucose metabolism [87] and changes in motor task-induced activation in ALS [88]. Additionally, ligand PET has provided *in vivo* evidence for activated microglia in ALS in motor and frontal lobe regions [89, 90], widespread reductions in flumazenil binding [91] in keeping with loss of inhibitory neuronal influences [92], and profoundly reduced binding of a ligand with serotonin receptor affinity in frontotemporal regions [93], also seen in FTD [94]. An emerging group of radiotracers targeting glutamate receptors holds particular promise for exploring the role of excitotoxic mechanisms in ALS pathogenesis [95], with evidence from the transgenic mouse model [96].

To date there have been no longitudinal PET studies in ALS.

#### MRS

Investigations of metabolic tissue content by means of MRS have consistently revealed NAA [97, 98] and GABA reductions in the primary motor cortex in ALS [99], as well as glutamate and glutamine increases in the brainstem [100]. Methodological variability continues to limit the wider diagnostic application of MRS.

### Imaging for Stratification

Several studies reported regional gray and white matter changes in relation to clinical and neuropsychological features, most commonly in relation to site of onset (limb *vs* bulbar), which is a well-established prognostic marker [101]. Investigation of VBM and TBSS correlates in a relatively large cohort of patients with sporadic ALS revealed that a higher burden of UMN involvement clinically was associated with a significant decrease of FA in the CSTs with co-localized increases in AD, as well as an increase of MD and RD in the CSTs, superior longitudinal fascicles, and in the corpus callosum [50], particularly in those with predominant UMN involvement [55, 102, 103]. Furthermore, surface-based analysis of the precentral gyrus revealed that compared with patients with ALS, patients with a UMN phenotype displayed reduced cortical thickness [70]. The involvement of extramotor cortical regions is more severe in patients with ALS with cognitive impairment and ALS-FTD [58].

Investigating MRI correlates of ALSFRS subscores in more detail, lower limb subscore correlated negatively with MD in the right CST, while VBM-based gray matter volume within the left primary motor cortex [50], as well as cortical thickness of corresponding body regions of the motor homunculus [70], were shown to be correlated with bulbar disability subscore. In addition, another study found bulbar-onset ALS to be associated with greater central white matter degeneration than limb-onset ALS [104]. VBM analysis has also revealed negative correlations between progression rate and left primary motor cortex volume [50], while TBSS analysis consistently demonstrated correlation of CST FA with progression rate [105].

While these cross-sectional findings suggest that there may be a range of neuroimaging marker signatures linked to different ALS phenotypes, imaging markers at present do not appear to offer added value to clinical assessment for the stratification of patients with ALS in the context of a clinical trial.

### Imaging Biomarkers of Disease Progression

#### Structural MRI Changes

Relatively few longitudinal MRI studies have been conducted in ALS (Table 1). Inconsistencies seem likely to reflect patient heterogeneity and variable interval between scans (compounded by small group sizes in some cases), plus differences in MRI acquisition and analysis protocols. Six published longitudinal studies focused solely on DTI changes over time. Two reported no DTI changes in the brain associated with disease progression (in both cases, DTI data was acquired at 1.5 T). One of these negative studies assessed changes in FA and MD along the CST using a ROI approach in 11 patients with ALS and 11 controls at 2 time points that were in some cases more than 6 months apart (intervals were variable, though matched between the 2 groups). While mean ALSFRS-R scores in the patient group changed significantly between examinations, UMN burden score remained qualitatively stable, and neither FA nor MD changed significantly over time [106]. The second study that reported negative results assessed cross-sectional area, FA and MD of the cervical cord, as well as CST average FA and MD in 17 patients with ALS at baseline and after a mean interval of 9 months. While all examined spinal cord metrics changed significantly, CST measures remained stable over time in patients with ALS [107]. The positive spinal cord results were in line with another more recent longitudinal study in which 14 patients underwent 2 MRI scans approximately 11 months apart [108]. At both time points the cross-sectional area of the cervical and upper thoracic spinal cord was measured, as well as FA, axial/radial/mean diffusivities, and magnetization transfer ratio within the lateral CST in the cervical region [108]. Cross-sectional area and magnetization transfer ratio changed significantly between baseline and follow-up, and the cross-sectional area rate of change was highly correlated upper limb ALSFRS-R subscore rate of change.

**Table 1:** Longitudinal neuroimaging studies in amyotrophic lateral sclerosis (ALS)

Reference	Field strength	n*	Method	Interval between scans	ALSFRS-R baseline–follow-up	Main results
[106]	1.5 T	11	DTI FA and MD in CST ROIs	~6 months	40–35	No significant changes
[107]	1.5 T	17	CSA/FA/MD in cervical cord, average FA and MD in CST	9 months	27–21	All metrics in the spinal cord, but not in the CST, changed significantly
[108]	3 T	14	Spinal cord CSA, FA, L1, RD, MD, and MTR in cervical region of lateral CST	11 months	40–31	Significant CSA and MTR changes
[49]	3 T	16	DTI tractography of CST, VBM using whole-brain FA maps	6 months	42 - 38	FA decreases in CST and CC
[110]	3 T	17	ROI analysis based on DTI tractography of CST, VBM of whole-brain FA and MD maps	8 months	35–29	FA decreases in right superior CST, MD stable
[111]	1.5 T	15	VBM using FA and ADC maps	6 months	35–33	FA decreases in CST, frontal areas, and cerebellum
[105]	3 T	19	TBSS of FA, MD, L1, RD	6 months	34–30	L1 increases in posterior limb of left internal capsule
[113]	1.5 T	16	TBM analysis of gray matter	9 months	27–21	Progression of atrophy in left premotor cortex and right putamen and caudate
[68]	3 T	20	Surface-based CT analysis	3–10 months	42–37	No significant changes
[114]	3 T	51	Surface-based CT analysis	7.8 months	39–33	No significant changes
[115]	3 T	39	Volumetry of subcortical gray matter and ventricles	5.5 months	41–36	Shrinkage of right CA 2/3, and CA 4/dentate gyrus; enlargement of both lateral ventricles and right third and fourth ventricle
[56]	3 T	17	VBM of gray matter structure and FA and MD	6 months	37–32	Widespread gray matter decreases, FA and MD changes in right cerebral peduncles
[116]	3 T	9	Gray matter CT, regional brain volumes, FA and CSA of CST and CC	1.3 years	40–34	CT and volume decreases of precentral gyri. FA stable, but CST CSA declined
[50]	3 T	27	VBM and TBSS of FA, MD, L1, and RD	>6 months	35–28	Widespread gray matter volume decreases, minor L1 and MD increases in CC, minor L1 increases in left CST
[117]	3 T	34	VBM and CT, volumetry of subcortical gray matter, average FA, MD, L1, and RD in CST ROI (intersection of TBSS skeleton and CST mask)	6 months	40–35	CST FA decreases, no gray matter changes
[118]	1.5 T	9	^1^H MRS: NAA, Cre, and Cho in motor and nonmotor regions	1 months, 3 months	–	NAA/Cre and NAA/(Cre + Cho) decreases in motor cortex after 1 month; absolute NAA, Cre, and Cho decreases after 3 months
[119]	1.5 T	28	^1^H MRS: NAA, Cre, and Cho in motor and nonmotor regions	Every 3 months for up to 12 months	–	NAA, Cre, and Cho decreases in motor cortex at 3 months but bot beyond
[120]	1.5 T	8	^1^H MRS: NAA, Cre, Cho, myoinositol, glutamate, and glutamine in motor cortex and white matter, including pyramidal tracts	3 months, 6 months	25–21– 18	NAA decreases in motor cortices between baseline and 6 months (and baseline and 3 months for less-affected hemisphere), NAA/(Cr + Cho) ratio decreases from baseline to 3 months, and from 3 to 6 months

ALSFRS-R = Amyotrophic Lateral Sclerosis Functional Rating Scale – Revised; DTI = diffusion tensor imaging; FA = fractional anisotropy; MD = mean diffusivity; CST = corticospinal tract; ROI = region of interest; CSA = cross-sectional area; L1 = axial diffusivity; RD = radial diffusivity; MTR = magnetization transfer ratio; VBM = voxel-based morphometry; ADC = apparent diffusion coefficient; TBSS = tract-based spatial statistics; TBM = tensor-based morphometry; CT = cortical thickness; CA = cornu ammonis; CC = corpus callosum; ^1^H MRS = proton magnetic resonance spectroscopy; NAA = *N*-acetylaspartate; Cre = creatine; Cho = choline. *Number of patients with ALS studied longitudinally

Four longitudinal DTI studies suggested cerebral DTI metrics to be sensitive to disease progression in ALS [49, 105, 109–111]. A combined 3-T diffusion tensor tractography of the CST and whole-brain voxel-based analysis of FA maps was used to investigate the sensitivity of these techniques to detect white matter changes in 16 patients after 6 months [49]. FA averaged across both CSTs was found to have significantly decreased only in the subset of limb-onset ALS. Looking at more detailed FA profiles along the CST between the 2 time points they found that in bulbar-onset ALS, FA decreased significantly over time in the cerebral peduncle/caudal part of the posterior limb of the internal capsule and in the subcortical white matter, while in limb-onset ALS, FA decreased significantly over time in the medulla oblongata, cerebral peduncle/caudal part of the posterior limb of the internal capsule, and in the subcortical white matter. Additionally, a whole-brain voxel-wise comparison of baseline and follow-up FA maps revealed extensive FA reduction in patients with bulbar onset of symptoms, comprising the white matter underneath the primary motor and sensory cortex, premotor cortex, inferior frontal gyrus, and dorsolateral prefrontal cortex, as well as the body and genu of the corpus callosum, the left thalamus, the hippocampal formations, and the right cingulum. In patients with limb-onset ALS, FA reduction over time was found throughout the CST, in the body of the corpus callosum, in the white matter underneath primary sensory cortex and anterior temporal pole, the right thalamus, and cingulum, as well as the left optic radiations. Reassuringly, none of the reported analysis approaches revealed any FA changes over time in 11 control subjects that were included in this study.

In a similar study, 3-T tractography of the CST to guide ROI analysis, as well as voxel-wise analysis, was used to assess FA and MD changes in 17 patients with ALS over an average interval of 8 months [110], with a significant decrease of FA, but not MD, in the right superior CST. This finding was corroborated by results of the whole-brain voxel-wise analysis. In another 1.5-T voxel-wise study, FA and apparent diffusion coefficient in 15 patients with ALS followed up after 6 months found FA reductions in the CST, frontal areas, and in the cerebellum, but with an associated nonsignificant average ALSFRS-R decline [111]. Using tract-based spatial statistics of FA and MD, as well as RD and AD metrics at 3 T in 19 patients, demonstrated a significant increase in AD in the posterior limb of the left internal capsule after a 6-month interval [105]. No FA, MD, or RD changes over time were detected in this study.

A graph theory, network approach to diffusion-weighted data was used with a group of patients with ALS studied twice with an interval of 5.5 months. This demonstrated an expanding disintegration of frontal and parietal connections to the primary motor cortex, supporting the spread of ALS pathology via structural connections [112]. This type of analysis is not yet quantifiable to an extent that would be meaningful in the context of a therapeutic trial.

Four published longitudinal MRI studies focused solely on the assessment of gray matter structural changes in ALS over time. A tensor-based morphometry study at 1.5 T in 16 patients and 10 healthy controls investigated ALS-related gray matter atrophy over an average interval of 9 months [113], and detected a significant progression of atrophy in the left premotor cortex and in the right putamen and caudate nucleus. A surface-based cortical thickness study in 20 patients with ALS did not find any significant changes over 3 to 10 months [68]. This negative result was corroborated by a similar study in a larger cohort of 51 patients with classic ALS, also showing no significant progression of cortical thinning over an average interval of 8 months [114]. Data for both studies were acquired at 3 T.

The change in the volume of subcortical gray matter structures and ventricles in 39 patients with ALS was assessed over an interval of on average 5.5 months [115], and significant shrinkage of the right cornu ammonis 2/3 and cornu ammonis 4/dentate gyrus was observed, as well as enlargement of both lateral, the right inferior lateral, and third and fourth ventricles.

Only 4 studies to date have investigated both white and gray matter structural decline in the same cohorts. Widespread volume decreases in gray matter were observed, particularly in the bilateral frontal and temporal lobes, in a combined VBM and DTI analysis with 6-month follow-up in 17 patients with ALS [56]. Additionally, white matter volume reductions and FA and MD changes were found near CST regions and, most prominently, in the right cerebral peduncles of the midbrain.

Examination of the longitudinal changes of cortical thickness, regional brain volumes, and DTI of the CST and callosum was undertaken in 9 patients with ALS over an average interval of 1.26 years [116]. Only those imaging measures that differed from controls in cross-sectional analyses were used as ROIs for longitudinal analyses. The results indicated that cortical thinning and gray matter volume loss of the precentral gyri progressed between baseline and follow-up. FA of the CSTs remained stable, but the cross-sectional area declined. Changes in clinical measures furthermore correlated with changes in precentral cortical thickness and gray matter volume.

In MRI acquired from 27 patients with sporadic ALS at 2 different time points at least 6 months apart [50], TBSS analysis of DTI data showed only limited significant increases for AD and MD in a corpus callosum ROI, as well as minor AD increases in the left CST. VBM analysis revealed widespread gray matter volume decreases in motor and frontotemporal regions, the thalami, and caudate heads bilaterally. These findings were in stark contrast with the results of a subsequent study of 3 time points over a mean total interval of 6 months in 34 patients with ALS [117]. While neither VBM, nor cortical thickness analysis or volumetry of deep gray matter structures indicated progressive gray matter atrophy, FA of the CST showed a significant linear decline over time but was still less sensitive than the ALSFRS-R for monitoring disease progression.

#### MRS

Only 3 MRS studies have investigated changes over time. One early study acquired structural MRI and multislice ^1^H MRSI data in 9 patients, with a minimum of 3 sequential measurements in order to obtain concentrations of NAA, Cre, and Cho in the left and right motor cortex, and in gray matter and white matter of nonmotor regions in the brain [118]. For the most affected motor cortex, NAA/Cre and NAA/(Cre + Cho) ratios decreased significantly after 1 month. After 3 months, absolute values of NAA, Cre, and Cho decreased significantly, while the observed metabolite ratio changes were not significant. For the least affected motor cortex, no significant changes were found over 3 months. Furthermore, while NAA, Cre, and Cho concentrations decreased over time in the motor cortex, concentrations in nonmotor regions remained qualitatively unchanged.

In a follow-up study by the same group, 13 patients with El Escorial criteria possible/suspected ALS and 15 patients with probable/definite ALS received repeated multislice ^1^H MRSI scans every 3 months for up to 12 months [119]. While again the NAA ratios [NAA/Cre, NAA/Cho, and NAA/(Cre+Cho)] did not show any significant longitudinal change, a decrease in NAA concentration was observed both within and outside the motor cortex in the more affected hemisphere only when comparing the baseline scan to 3-month follow-up data, but not beyond. Similarly, Cre and Cho decreases were observed in the motor cortex of the more affected hemisphere, while in frontal and parietal nonmotor regions no changes were observed for Cre at 3 months, but decreases were observed for Cre at 9 months in the more affected hemisphere. Cho decreases were observed at all time points in the more affected hemisphere when compared with the initial baseline scan, and in the less affected hemisphere at both the 6- and 9-month time points.

A third study assessed ^1^H MRS data of the gray matter of the motor cortex and the white matter, including the pyramidal tracts, and investigated concentrations of NAA, Cr, Cho, myoinositol, glutamate, and glutamine in 8 patients with definite ALS at 3 time points (baseline, and after 3 and 6 months) [120]. In line with the results of the 2 previous studies the patient group also showed a significant NAA decline in the motor cortex of both of the clinically more and less affected hemispheres between first measurement and month 6, as well as between baseline and month 3 for the less affected side. For the NAA/(Cr + Cho) ratio, significant decline in the less affected hemisphere was observed between baseline and month 3 and to month 6, as well as from month 3 to month 6. In white matter regions, however, neither NAA nor the NAA/(Cr + Cho) ratios changed over time.

Taken together these longitudinal imaging findings in ALS suggest that white-matter pathology may be significantly established in the symptomatic phase of ALS where therapeutic trials are most likely to take place, with the magnitude of change likely then greatest in those with the fastest rates of disease progression.

## Neuroimaging Biomarkers Applied to Therapeutic Trials

### Applications in Other Neurodegenerative Disorders

The Alzheimer’s Disease Neuroimaging Initiative has been pivotal in developing the potential of neuroimaging outcome measures in Alzheimer’s disease therapeutic trials [121]. Significant challenges remain, particularly in the desire to include functional measures in Alzheimer’s disease [122] and now also in Parkinson’s Disease [123]. MRI was included as an exploratory surrogate marker in a trial of creatine in those at risk of Huntington’s disease, in which a treatment-related slowing of cortical atrophy was demonstrated [124]. Many of the challenges to the implementation of neuroimaging endpoints in neurodegenerative disorders, including ALS, are generic.

### Limitations of Clinical Measures

Clinical outcome measures in neurodegenerative disorders generally do not enable the distinction between disease-modifying and symptomatic drug effects. Symptoms might temporarily improve under the influence of a drug without a change in the actual disease activity, which could lead to an initial overestimation of a potential beneficial drug effect. Disease-modifying effects might also be missed during the trial period if they are not immediately reflected in a symptomatic response. Clinical scores are not objective and may be far removed from the underlying histopathological changes. They may be influenced by factors not directly disease-related and cannot be controlled, such as patient motivation and unwanted drug side-effects, which can result in reduced statistical power to detect therapeutic effects and consequently necessitate larger test cohorts. It has also been noted that rate of progression as defined by ALSFRS-R in patients seen soon after symptom onset is less reliable [12], so that neuroimaging may have a particular value as an objective marker in this group. Finally, the natural redundancy of biological systems suggests that clinical symptoms become evident at a relatively advanced stage of pathology. Clinical scores, by definition, are not able to assess any neuroprotective therapies applied to presymptomatic stages.

### Power Estimates for the Detection of Therapeutic Effect

Few longitudinal imaging studies in ALS have attempted to estimate the power of MRI in the detection of therapeutic effect. Changes of FA rates in the right superior CST were used to estimate a sample size of 263 patients per arm (placebo and treatment) to detect a hypothetical treatment effect size of 25% with 80% power [125]. Another study used the significant annual rate of change for both ALSFRS-R and FA with the conclusion that the sample size needed would be 94 patients per arm for ALSFRS-R *versus* 567 for FA [117].

### Safety and Artifacts

Potential safety risks associated with different brain imaging methods are an important factor that requires consideration before application in clinical trials [126]. MRI does not involve ionizing radiation and is generally considered harmless. However, the strong magnetic field involved necessitates careful screening for contraindications, as it will attract ferromagnetic objects and can cause them to move suddenly with great force. In some cases even nonmagnetic medical implants can move, and implants, as well as tattoos, can heat substantially during an MRI scan, potentially causing injury to the examined patient. Metallic implants, dentures, fillings, permanent makeup, and so on, can furthermore cause image distortion, rendering them useless for some analyses. More specific to ALS, some patients struggle to tolerate long scanning sessions in a supine position due to joint discomfort, orthopnea, or oral secretion control. The level of radiation exposure inherent to PET is relatively low in single study but becomes an additional concern with repeated measurement.

### Robustness, Reproducibility, Quantification, and Harmonization

Before an imaging marker can be proposed as a useful outcome measure in a clinical trial, the *robustness* and *reproducibility* of the respective technique or analysis method must be evaluated. While in every imaging study the raw data have to pass some degree of initial quality control to exclude nondisease-related gross anatomical abnormalities and artifacts (such as visual inspection by an experienced image analyst), some postprocessing algorithms require higher degrees of data quality than others in order to be able to run successfully. In research studies, data are often simply classified as not analyzable and discarded when a certain algorithm fails. However, this exclusion of data in the research setting may lead to a biased assessment of the “real-world” usefulness as a biomarker, as an algorithm that appears to be very sensitive for the detection of disease-related abnormalities, but necessitates the exclusion of some data or subjects, might ultimately require larger test cohorts if used as an endpoint in a clinical trial.

Another factor is the generalizability of outcomes derived from research studies that tend to be based in a single imaging center, often utilizing highly advanced image acquisition hardware and image analysis software and expertise not easily available in a clinical setting. In contrast, most advanced therapeutic trials are run in a multicenter setting, posing the problem of combining data from a variety of scanners, potentially acquired with different imaging sequences that are dictated by scanner capabilities and limited by hardware constraints. Usage of multiple scanners, as well as the fact that even on the same scanner performance is likely to change over time, necessitates harmonization of data acquisition and analysis protocols between centers and, most importantly, stringent quality control. For instance, phantom measurements can be incorporated to assess both scanner performance and the validity of postprocessing image corrections in order to reduce systematic errors in human data [127]. This should, in turn, increase statistical power for the assessment of brain changes during therapeutic trials.

It is possible to assess systematic differences across centers when applying a harmonized acquisition protocol [128], and then to implement a common database [129]. A feasibility study involving data from 8 international ALS-specialist clinic sites, as part of the Neuroimaging Society in ALS (www.nisals.org), retrospectively combined DTI FA maps of control participants to establish correction matrices, with correction algorithms then successfully applied to the FA maps of the control and ALS patient groups [130]. To establish a sense of the overall degree of reproducibility of the imaging metrics of interest and to be able to distinguish normal intraindividual variability from changes due to ageing, disease progression, or drug effects, a cohort of healthy individuals should be scanned repeatedly over a short period of time at each scanner site. This is particularly important as even metrics usually labeled as quantitative, such as FA, are influenced by hardware performance fluctuations. Lastly, care should be taken to scan an equal number of patients and controls at each site and close in time, to ensure similar levels of subject hydration at each scan [131], and use data preprocessing pipelines that are optimized for longitudinal analyses and do not introduce biases during intrasubject image registration [132].

### Disease-Specific Effects *versus* Epiphenomena

An important caveat for the application of fMRI in search of potential therapeutic targets and markers of disease progression is that part of the observed differences between patients and controls, as well as changes over time revealed by imaging in ALS, might simply reflect secondary changes caused by clinical symptoms. These might be maladaptive or compensatory. Results of ongoing prospective imaging studies in large cohorts of randomly selected healthy people (e.g., http://imaging.ukbiobank.ac.uk), or select individuals with a high genetic risk of developing ALS in the future that have not yet developed symptoms (e.g., Pre-fALS study, University of Miami, FL, USA) may help distinguish disease-related brain abnormalities from symptom-related changes and identify neuroprotective targets.

### Ageing Confounds

To be able to quantify potential beneficial drug effects on the brain, the brain changes that occur naturally as the disease progresses need to be known first. As disease progression and ageing are invariably correlated, complementary longitudinal brain imaging studies in healthy volunteers are optimal. Owing to limited resources and ethical considerations (e.g., PET studies in healthy individuals), only single time-point data have generally been acquired for healthy controls in the research setting.

### Drug Effects on Therapeutic Functional Targets *versus* Global Effects

While BOLD fMRI has the advantage of being a noninvasive technique that bypasses some of the safety concerns related to the use of radioactive substances used in PET, it comes with 1 major limitation that must be considered when interpreting the BOLD signal, particularly in the context of pharmacological research. Besides only providing an *indirect* measurement of neuronal activity, BOLD fMRI can only detect *changes* in blood flow, and does not provide information about the absolute amount of CBF. Consequently, if a drug’s effects on neurovascular coupling are unknown, a change in the BOLD signal might not only reflect its influences on neural activity, but also its potential effects on neuronal signaling to the blood vessels involved in the CBF response, or on vasculature itself. Inclusion of healthy volunteers or implementation of complementary techniques, however, can help to discern neural activity and vascular components of the BOLD signal. MR-based perfusion techniques, such as arterial spin labeling can be used to obtain quantitative CBF maps either as a single baseline image or in a time series, in order to control for global drug-related CBF changes. Arterial spin labeling fMRI is, however, technically challenging and suffers from poorer signal-to-noise ratio, and lower spatial and temporal resolution than BOLD. An alternative approach that can assist interpretation of the BOLD signal is direct quantitative measurement of neural activation using electrophysiological techniques, such as electroencephalography or magnetoencephalography [133, 134]. Both techniques offer excellent temporal resolution in the millisecond range, but magnetoencephalography offers superior spatial resolution with the caveat of currently being available only in specialist centers.

## Conclusions and Future Directions

MRI is unique in being able to assess simultaneously brain structure and function, and provide a deeper understanding of *in vivo* evolution of cerebral pathology as a link between cellular and system dysfunction in ALS. DTI metrics are promising diagnostic biomarkers, able to identify brain white matter abnormalities closely linked with known ALS pathology, and may have a particular niche in the detection of occult UMN involvement thereby expanding the trial inclusion pool. Inconsistent findings resulting from longitudinal studies in ALS can be substantially attributed to small and clinically heterogeneous groups, and analysis methodology for some sequences, rather than insensitivity of MRI to structural and functional cerebral pathology. Ultimately, a multimodal approach combining imaging, neurophysiology, and biofluid markers may be needed to provide a signature sensitive enough to stratify and measure the effect of therapeutics on disease activity at the level of the individual patient. Meanwhile, postmortem MRI studies systematically comparing diffusion and other structural imaging metrics with histopathological markers [135] may shed more light on the pathological relevance of various MR signals at the histological level, including emerging hypotheses around spread of pathology [136], and so provide equally important mechanistic clues to guide therapeutic targeting.

## Electronic supplementary material

Below is the link to the electronic supplementary material.

ESM 1 (PDF 1224 kb)
